# Between prescription and deprescription: a qualitative study on the trajectory and challenges in the use of benzodiazepines among older adults

**DOI:** 10.3389/fphar.2026.1826743

**Published:** 2026-05-14

**Authors:** Silvio José Elisei Carvalho Junior, Marlon Silva Tinoco, Aline Istefane de Camargos Ramos, Luciana Soares Rodrigues, Denise Alves Guimarães, André Oliveira Baldoni, Mariana Linhares Pereira

**Affiliations:** 1 Universidade Federal de São João del-Rei, Divinópolis, Minas Gerais, Brazil; 2 Centro Universitário de Formiga, Formiga, Minas Gerais, Brazil

**Keywords:** benzodiazepines, clinical protocols, deprescription, medicalization, qualitative research

## Abstract

**Introduction:**

In Brazil, benzodiazepines rank among the twenty most prescribed medications in primary healthcare. Beyond the lack of robust evidence supporting chronic use, there has been a growing utilization of this drug class for managing conditions that were previously not formally diagnosable or treatable, such as secondary insomnia and bereavement experiences.

**Objective:**

In the context of benzodiazepine deprescription, the aim was to understand the trajectory of benzodiazepine use among older adults treated in Primary Health Care, identifying reasons for use and expectations related to its maintenance in pharmacotherapy.

**Method:**

An exploratory qualitative study was conducted through semi-structured interviews with twenty-nine individuals aged 60 years or older who had been using benzodiazepines for at least 1 month. Data were recorded in a field diary and interviews were audio-recorded. Subsequently, the data were analyzed using thematic analysis.

**Results:**

From the data analysis, two thematic categories emerged that together describe the trajectory of benzodiazepine use: the reasons related to initiation and the difficulties in discontinuation. In the first category, core meanings such as insomnia, life events, and work-related illness were identified, while in the second category the expectations regarding medication use stood out, reinforcing continuity and hindering deprescription.

**Conclusion:**

Prescriptions were often not clinically justified or were based on a merely medicalizing logic. Furthermore, the deprescription process was found to be extremely complex for health professionals, as it is influenced by a series of biopsychosocial barriers.

## Introduction

In 1955, chemist Leo Sternbach developed the first benzodiazepine (BZD), marketed from 1960, and by 1990, approximately 18 million prescriptions had already been issued worldwide (Wick, 2013). Originally indicated for psychiatric and neurological disorders, its use extended to non-medical conditions such as stress, mild anxiety, and “nervousness,” illustrating the medicalization process described by Foucault and Conrad (Wick, 2013; Conrad, 2007; Foucault and Machado, 1984). Foucault traces the origins of medicalization back to the 17th century, conceptualizing it as a tool of state biopower that regulates bodies in the name of social health, encompassing processes from the indoctrination of gender roles to the psychiatrization of deviant behaviors (Foucault and Machado, 1984). In this sense, medicalization not only expanded the scope of medical practices but also legitimized the incorporation of psychotropic agents such as benzodiazepines into mental health management (Nappo and Carlini, 1993). In Brazil, this process is evident in routine clinical practice, where benzodiazepines, initially intended for specific psychiatric disorders, have become widely prescribed for common conditions such as insomnia and mild anxiety, thereby reinforcing the logic of medicalization by transforming social and emotional experiences into objects of pharmacological intervention (Foucault and Machado, 1984; Nappo and Carlini, 1993; Brazil. Ministry of Health, 2018; Madruga et al., 2019).

In Brazil, despite regulatory measures to prevent inappropriate prescribing—such as the requirement of special authorizations across the entire chain of production, distribution, and commercialization—the country remains the third largest consumer of benzodiazepines worldwide (Brazil. Ministry of Health, 2018; Madruga et al., 2019). Moreover, this drug class is among the twenty most used in Primary Care, disregarding solid evidence regarding the risks of chronic use (Oliveira et al., 2021). Its adverse effects include anterograde amnesia, cognitive decline, daytime sleepiness, dependence, tolerance, and increased risk of falls, reinforcing the need for rational prescription and eventual deprescription when the risk-benefit ratio is unfavorable, as is the case for older adults who, due to metabolic and physiological adaptations, are more susceptible to these events (Freire et al., 2022; Pottie et al., 2018).

Nevertheless, although dose reduction or drug discontinuation may bring benefits, biopsychosocial barriers such as intolerance to suffering, socioeconomic limitations, and expectations regarding the drug’s effects may hinder deprescription (Andrade et al., 2012). In addition, unequal access to mental health services and the scarcity of non-pharmacological alternatives exacerbate the problem (Rossi et al., 2020). These barriers highlight the urgency of developing multidimensional approaches that consider both the structure of health services and the social and cultural determinants in BZD deprescription (Araujo, 2021).

Currently, Primary Health Care (PHC) in Brazil is organized according to the logic of the Family Health Strategy (FHS), which contrasts with the biomedical model by emphasizing decentralized surveillance, community bonds, and continuity of care (Pulhiez and Norman, 2021). PHC is composed of general practitioners, multidisciplinary teams, and pharmacies linked to the public system, functioning as the gateway to controlled medications. This arrangement is integrated into the Unified Health System (SUS), a universal and free public policy that structures access to healthcare in Brazil and ensures comprehensive coverage for the entire population. The biomedical model, in turn, is characterized by reducing human suffering to pathophysiological alterations and by adopting a disease-centered approach, in which medications are regarded as the sole therapeutic alternative (Rossi et al., 2020; Araujo, 2021; Pulhiez and Norman, 2021). In this sense, such rationale fosters medicalization by transforming social and emotional experiences into objects of pharmacological intervention. In contrast, the FHS seeks to overcome this perspective by proposing expanded, person-centered practices, creating strategic opportunities to reorient drug therapy, reduce the excessive use of psychotropic agents, and promote deprescription (Pulhiez and Norman, 2021). In this context, the aim was to understand the trajectory of benzodiazepine use among older adults treated in PHC, identifying reasons for use and expectations related to the maintenance of these medications.

## Objective

In the context of benzodiazepine deprescription, the aim was to understand the trajectory of BZD use among patients aged 60 years or older treated in Primary Health Care (PHC), identifying reasons for use and expectations related to the maintenance of these medications.

## Methods

### Study design

Exploratory qualitative study, part of the umbrella project “Effect and feasibility of implementing a benzodiazepine deprescription protocol.” It was conducted in PHC in a municipality with an estimated population of 21,046 inhabitants in the interior of Minas Gerais (Brazilian Institute Of Geography And Statistics, 2026; Minayo, 2014).

### Ethical approval

This study was submitted to and approved by the Research Ethics Committee (CEP) involving human subjects of the Federal University of São João del-Rei (UFSJ), CAAE: 30688320.0.0000.5545.

### Participant recruitment

Participants were identified and referred by managers and community health agents (CHA) from the Family Health Strategy (FHS), according to the criteria of benzodiazepine use for at least 1 month without current clinical justification, defined as situations supported by clinical guidelines, including severe anxiety disorders, refractory insomnia, alcohol withdrawal syndrome, and epilepsy. Individuals aged 60 years or older and receiving follow-up care within the Unified Health System (SUS) were included (Pottie et al., 2018). Selection was carried out through convenience sampling, specifically considering medication use, age group, and the proximity of CHAs to users, a factor that may limit the generalizability of the findings. Many patients maintained use solely through automatic prescription renewals, without new clinical evaluation (Camargo et al., 2023). Those requiring continuous use and those without adequate cognitive conditions for participation were excluded. Of the 30 identified, only one declined the interview.

### Interview and data collection procedures

Data collection was conducted through interviews guided by a semi-structured script composed of three parts: participant characterization, self-reported health conditions, and pharmacotherapeutic variables (Minayo, 2014). In the final stage, participants were invited to join the deprescription project, report their BZD use trajectory, and reasons for accepting or declining the invitation. Interviews, conducted between 23 May 2023, and 12 December 2023, mostly took place at participants’ homes and lasted an average of 30 min.

### Data triangulation

Information was collected using the technique of data triangulation, which involves combining different methods of data collection in order to compare results and strengthen the understanding of the phenomenon. In this study, the data sources included interviews (recordings), field notes, and participant observation (Minayo, 2014).

After signing the informed consent form (ICF), the interviews were audio-recorded for subsequent transcription. Field notes were also employed as a tool to document observations not captured by the recordings and aspects of the construction of intersubjectivities, thereby providing access to deeper layers of social reality (Morse and Field, 1995; Mays and Pope, 1995; Cachado, 2021; Bardin, 2011).

### Data analysis

Among the 29 interviewees, only one participant did not consent to having the interview recorded (E26); however, the field notes were taken into account. The interviews were recorded using the Voice Recorder software, available on the Google Play Store, and the transcriptions were manually performed in Microsoft Word. The information from the field notes was also fully transcribed and analyzed through thematic or categorical analysis, following three stages: the pre-exploration phase or floating reading, which involves reading and preparing the material; the search and selection of meaning units/coding, consisting of the identification of emerging themes; and finally, the categorization of units and inferences (Bardin, 2011). In this study, a meaning unit was defined as any excerpt of discourse that expressed a complete idea related to the object of investigation, serving as the basis for coding and subsequent categorization. No specific software was used for the analysis, as the entire process was conducted manually. To preserve participant confidentiality, an alphanumeric system was adopted to code the interviews, identified from E1 to E29.

### Results participant characterization

The sample consisted primarily of women and individuals who had not completed elementary education. Details of the characterization are presented in Table 1. Only five (E4, E7, E17, E21, and E26) of the twenty-nine interviewees refused to initiate the deprescription process.

**TABLE 1:** Profile of older adults interviewed who were using benzodiazepines.

Id	Age	Sex	Education level	Marital status	Duration of use	Self-reported indication
E1	76 years	Female	Incomplete primary	With partner	38 years	Bereavement
E2	63 years	Male	Incomplete primary	Widowed	30 years	Bereavement
E3	75 years	Female	Incomplete primary	Divorced	10 years	Insomnia
E4	76 years	Female	Complete secondary	Widowed	30 years	Bereavement/Insomnia
E5	75 years	Male	Incomplete primary	Married	40 years	Traumatic event
E6	72 years	Female	Incomplete primary	Married	40 years	Work-related illness (anxiety)
E7	98 years	Female	No schooling	Widowed	40 years	Bereavement
E8	68 years	Male	Complete primary	Divorced	5 years	Dependence
E9	66 years	Male	Complete primary	Married	5 years	Traumatic event (insomnia)
E10	73 years	Female	Incomplete primary	Married	14 years	Traumatic event (heart attack)
E11	71 years	Male	Incomplete primary	Married	22 years	Alcohol use
E12	76 years	Female	Incomplete primary	Married	20 years	Nocturnal polyuria
E13	73 years	Male	Incomplete primary	Married	15 years	Work-related illness (night shifts)
E14	64 years	Male	Complete primary	Married	2 months	Nocturnal polyuria
E15	84 years	Female	Higher education	Widowed	3 years	Insomnia
E16	76 years	Female	Complete secondary	Widowed	30 years	Bereavement/Insomnia
E17	72 years	Female	Incomplete primary	Divorced	1.5 years	Insomnia (traumatic event)
E18	78 years	Male	Incomplete primary	Married	30 years	Alcohol use
E19	73 years	Female	Incomplete secondary	Married	20 years	Bereavement
E20	74 years	Female	Incomplete secondary	Married	10 years	Traumatic event (depression)
E21	92 years	Female	Incomplete primary	Widowed	6 years	Bereavement
E22	66 years	Female	Incomplete primary	Divorced	20 years	Anxiety (cyst)
E23	77 years	Female	No schooling	Widowed	21 years	Bereavement
E24	77 years	Male	No schooling	Married	10 years	Insomnia
E25	77 years	Female	Incomplete primary	Married	31 years	Bereavement
E26	75 years	Female	Incomplete primary	Married	30 years	Doesn’t remember
E27	74 years	Male	Incomplete primary	Married	30 years	Nervousness (unspecified)
E28	80 years	Female	Incomplete primary	Married	7 years	Insomnia
E29	75 years	Female	Incomplete primary	Divorced	10 years	Insomnia

### Thematic categories

Data analysis revealed two thematic categories that, together, outline the trajectory of benzodiazepine use: reasons for initiation and challenges in discontinuation. The first category encompassed meaning units such as insomnia, life events, and work-related illness, while the second highlighted expectations surrounding medication use, which reinforce continuity and hinder deprescribing. Examples of interviewee statements were used to illustrate the meaning units. The categories and their respective meaning units are represented in Table 2.

**TABLE 2:** Thematic categories and their respective core meanings.

Thematic category	Core meaning
Reasons for initiating benzodiazepine use	Sleep-related problems
	Life events
	Work-related illness
	Substance dependence
Challenges in discontinuing benzodiazepine use	Expectations regarding medication effects

## Reasons related to the initiation of benzodiazepine use

### Sleep-related problems

When asked about the etiology from this condition, two interviewees reported that they could not sleep because they woke up frequently during the night to go to the bathroom, showing clear signs of nocturia. This statement can be illustrated by the following excerpts:

*E12: “[…] I sleep very poorly, I go to the bathroom all night […]”*

*E14: “[…] I woke up a lot to go to the bathroom… I woke up three times every day to urinate […]”*

During the interview, E14 reported that he had long suffered from severe glycemic dysregulation, which led to diabetic retinopathy. However, after consulting an endocrinologist, he showed significant improvement. Nevertheless, in a routine consultation with a cardiologist, the participant reported that many of his medications for diabetes were replaced, and his insulin dose was reduced. As a consequence, E14 reported that his hyperglycemia returned, accompanied by nocturia. Because of this, the participant began to have difficulty sleeping, which led him back to the cardiologist, who, faced with his “insomnia” caused by nocturia, prescribed a BZD.

A different case was presented by E3, who reported being unable to sleep mainly due to environmental factors that disrupted his rest:

*E3: “[…] I sleep with my grandson… the television stays on and it disturbs my sleep … I end up not being able to sleep… then you get used to the medication, right?! […]”*

### Life events

Many participants stated that they began treatment due to exposure to life events such as bereavement and traumatic experiences.

*E1: “[…] I started using it after my nephew was killed […]”*

*E19: “[…] I lost a 14-year-old daughter… I thought everything was lost … I had 10 children, now I only have 3 […]”*

*E5: “[…] I became sad after the heart attack […]”*

### Work-related illness

Work-related illness also had a strong influence on the initiation of treatment. Often, it was reported as a form of “stress” or anxiety generated by work:

*E6: “[…] I was very anxious at work […]”*

A different case was reported by E13, who stated that for many years he worked night shifts without a fixed schedule, waiting to be called. Because of this, he slept, but his sleep was not restorative.

*E13: “[…] I managed to sleep, but I woke up startled… I kept waiting for the phone to ring… because of that I unlearned how to sleep […]”*

### Alcohol withdrawal syndrome

Interviewees also reported that symptoms related to alcohol withdrawal were decisive factors for initiating treatment.

*E11: “[…] after I stopped drinking, I became depressed […]”*

### Difficulty in discontinuing benzodiazepine use

When invited to participate in the deprescription project, many participants stated that they could not stop using BZD, as they believed that without the medication they would not be able to sleep or carry out daily activities. They also reported that even when they did not “need” the medication, its mere absence or unavailability already made sleep difficult.

*E12: “[…] you have to know it is here […]”*

*E20: “[…] how am I going to sleep … If you think you do not take it, you do not sleep.*

*[…]” E22: “[…] if you do not have it, you think you will not sleep […]”*

*E23: “[…] sometimes you do not even need it, but if you do not take it, you do not sleep […]”*

In addition to the statements that it was necessary to “have the medication nearby,” other participants reported that, many times, it was necessary to use the medication because they had “become accustomed” to it.

*E15: “[…] oh my God… what do we do, because we get used to it … we cannot manage without it […]”*

*E19: “[…] I got used to the medication… I have to take it […]”*

At another point, one interviewee claimed that if she did not use the medication, she would not even be able to work.

*E17: “[…] if we do not take the medication in the morning, we are unable to work” […]”*

Frequently, the invitation to participate in the deprescription project generated indignation, fear, and, in some cases, resistance or more intense emotional responses, as observed in the statement of E26:

*E26: “[…] Rivotril you don’t even need to try to take away… if I go without it, I cannot eat at all […]”*

After this statement, researchers tried to explain the purpose of deprescription, but E26 responded:

*E26: “[…] you don’t even need to continue explaining… I will not go without it […]”*

## Discussion

### Reasons related to the initiation of benzodiazepine use

From the reports, it is observed that in many cases there was no clinical justification for BZD use, as the accounts indicated insomnia secondary to nocturia or environmental factors. In the case of withdrawal syndrome, prescription is usually given in a fixed-dose scheme, with reduction after 5–7 days, or in a rescue scheme. Thus, none of the cases presented justify BZD use. The major problem is that an approach centered on “insomnia” can mask underlying problems and expose patients to risks, as in E14’s case, where glycemic dysregulation persisted. In E12, nocturia possibly resulted from evening or nighttime use of diuretics, while in E14, from severe glycemic dysregulation (Fontaine et al., 2021; Whitaker, 2017). These reports show how focusing on the symptom can neglect underlying causes, illustrating Sancho et al.‘s (2019) critique of the biomedical model, in which “a disease name implies a drug name,” favoring the complaint-response model of care, where consultation is reduced to providing an immediate response to the patient’s complaint, silencing diagnosis through the overlay of medication.

Regarding life events, PHC professionals often face demands involving psychological suffering, such as anxiety and sadness (Minóia and Minozzo, 2015). However, many professionals struggle to provide adequate support, as current diagnostic criteria reinforce the idea that medication use is indispensable for treating these issues. Added to this is the scarcity of material resources, time, continuing education, and other support tools (Minóia and Minozzo, 2015; Cassiano et al., 2019). Furthermore, prejudice and stigma often lead to distant and resistant care, reinforcing prescription or automatic renewal of psychotropic prescriptions (Cassiano et al., 2019).

At another point, the interviews revealed the existence of a paradox between socioeconomic relations and the social being, mainly due to the precariousness of working conditions, new assignments within a reduced timeframe, and, above all, the narrowing of the space between work activity and private life. This was exemplified by E13, who reported that, after working for many years in night shifts without fixed hours, he slept in a fragmented and non-restorative manner, always on alert for the telephone, even stating that he had “unlearned how to sleep” (Antunes and Praun, 2015). This account demonstrates how structural and contextual factors, such as the precariousness of work, the absence of routine, and insecurity, permeate everyday life and produce suffering, encouraging the use of pharmacological solutions as a way to maintain the individual’s work capacity, even without support from official guidelines (Antunes and Praun, 2015; Conrad, 1992; Barboza et al., 2024; Araujo, 2021).

In the case of alcohol dependence, although BZD are considered first-line therapy, treatment should not be restricted only to physiological aspects, since dependence often reflects political, social, and economic problems that generate inequality and exclusion. Thus, a drug-centered approach can mask problems that require public debate and intervention, perpetuating the cycle of discrimination and dependence (Hall, 2007).

### Difficulty in discontinuing benzodiazepine use

The symbolism attributed to medications is not a recent phenomenon. On the contrary, as early as 1983 researchers such as Lefévre discussed the existence of an allegory surrounding medications. According to the author, there is a mercantile logic that places health as a commodity to be acquired and medication as the means to obtain it quickly and efficiently. This is justified by the fact that historically, when society entered the capitalist production universe, there was an intense diffusion of the idea that through the consumption of a given commodity, it would be possible to reduce the distance between the concrete and the abstract. In this sense, health began to operate as an abstract object of desire, which, through an organicist reduction, had the consumption of the commodity drug as its only response, ignoring the existence of any other determinants (Lefevre, 1991).

In the participants’ accounts, this logic is manifested in statements such as that of E26, who reported being unable even to eat without the use of medication, or E17, who stated that he could not work without “the morning pill.” It is observed that the use of this meaning, combined with a strictly medicalizing perspective, may lead the individual to create the expectation that the use of benzodiazepines would be the only solution to their distress (Conrad, 1992; Lefevre, 1991). The reluctance to initiate deprescription suggests that the symbolism attributed to the medication shapes expectations about its effects, reinforcing the perception of necessity and anticipating difficulties in discontinuing its use. The phenomenon of medicalization, in turn, reinforces this perception and leads the individual to believe that they need the drug, while also feeling insecure about stopping it (Conrad, 1992; Lefevre, 1991). In addition, the patients’ narratives revealed barriers such as fear of not sleeping, symbolic dependence on the medication, expectations of immediate relief, and socioeconomic limitations. These elements not only highlight individual difficulties but also indicate the context in which healthcare professionals operate, reinforcing the challenges faced in the deprescription process. According to Conrad, this is one of the consequences of excessive medicalization, a process in which non-medical problems are treated as medical ones (Conrad, 1992). The main limitation to be considered relates to the study population and context, which, being from a single municipality, may not reflect the reality of other locations. On the other hand, this study innovates by addressing the history of use and the difficulties faced in the context of implementing a BZD deprescription protocol in PHC.

The findings of this study reveal a considerable proportion of benzodiazepine prescriptions lacking clinical justification or issued solely based on patient-reported complaints. Furthermore, many interviewees lacked the resources to change their circumstances or to access psychotherapy. Therefore, it is necessary not only to review the criteria for promoting rational prescription, but also to develop a more flexible benzodiazepine deprescription protocol, applicable to different realities, in order to make its implementation feasible and contextually coherent, since, in most cases, the success of this process depends on the singularity and context of the individual and the health service.

Finally, it is essential to resume debates involving mental health within the scope of the Unified Health System (SUS), in order to strengthen existing public policies and, consequently, offer alternative forms of treatment beyond psychotropic medication.

## Data Availability

The original contributions presented in the study are included in the article/supplementary material, further inquiries can be directed to the corresponding author.
